# Deeply 3D-T1-TFE hypointense voxels are characteristic of phase-rim lesions in multiple sclerosis

**DOI:** 10.1007/s00330-023-09784-w

**Published:** 2023-06-06

**Authors:** Pablo Naval-Baudin, Albert Pons-Escoda, Àngels Camins, Pablo Arroyo, Mildred Viveros, Josep Castell, Mònica Cos, Antonio Martínez-Yélamos, Sergio Martínez-Yélamos, Carles Majós

**Affiliations:** 1 Neuroradiology Section, Department of Radiology, Hospital Universitari de Bellvitge, L’Hospitalet de Llobregat, Carrer de Feixa Llarga SN, 08907 Barcelona, Spain; 2 Institut de Diagnòstic Per La Imatge (IDI), L’Hospitalet de Llobregat, Centre Bellvige, Carrer de Feixa Llarga SN, 08907 Barcelona, Spain; 3 Bellvitge Biomedical Research Institute (IDIBELL), Universitat de Barcelona (UB), L’Hospitalet de Llobregat, 08907 Barcelona, Spain; 4 Departament de Ciències Clíniques, Facultat de Medicina I Ciències de La Salut, Universitat de Barcelona (UB), Carrer de Casanova 143, 08036 Barcelona, Spain; 5 Multiple Sclerosis Unit, Department of Neurology, Hospital Universitari de Bellvitge, L’Hospitalet de Llobregat, Carrer de Feixa Llarga SN, 08907 Barcelona, Spain

**Keywords:** Multiple sclerosis, Magnetic resonance imaging, Biomarkers, Susceptibility-weighted imaging, Phase-rim lesions

## Abstract

**Objectives:**

The development of new drugs for the treatment of progressive multiple sclerosis (MS) highlights the need for new prognostic biomarkers. Phase-rim lesions (PRLs) have been proposed as markers of progressive disease but are difficult to identify and quantify. Previous studies have identified T1-hypointensity in PRLs. The aim of this study was to compare the intensity profiles of PRLs and non-PRL white-matter lesions (nPR-WMLs) on three-dimensional T1-weighted turbo field echo (3DT1TFE) MRI. We then evaluated the performance of a derived metric as a surrogate for PRLs as potential markers for risk of disease progression.

**Methods:**

This study enrolled a cohort of relapsing–remitting (*n* = 10) and secondary progressive MS (*n* = 10) patients for whom 3 T MRI was available. PRLs and nPR-WMLs were segmented, and voxel-wise normalized T1-intensity histograms were analyzed. The lesions were divided equally into training and test datasets, and the fifth-percentile (p5)-normalized T1-intensity of each lesion was compared between groups and used for classification prediction.

**Results:**

Voxel-wise histogram analysis showed a unimodal histogram for nPR-WMLs and a bimodal histogram for PRLs with a large peak in the hypointense limit. Lesion-wise analysis included 1075 nPR-WMLs and 39 PRLs. The p5 intensity of PRLs was significantly lower than that of nPR-WMLs. The T1 intensity-based PRL classifier had a sensitivity of 0.526 and specificity of 0.959.

**Conclusions:**

Profound hypointensity on 3DT1TFE MRI is characteristic of PRLs and rare in other white-matter lesions. Given the widespread availability of T1-weighted imaging, this feature might serve as a surrogate biomarker for smoldering inflammation.

**Clinical relevance statement:**

Quantitative analysis of 3DT1TFE may detect deeply hypointense voxels in multiple sclerosis lesions, which are highly specific to PRLs. This could serve as a specific indicator of smoldering inflammation in MS, aiding in early detection of disease progression.

**Key Points:**

• *Phase-rim lesions (PRLs) in multiple sclerosis present a characteristic T1-hypointensity on 3DT1TFE MRI.*

• *Intensity-normalized 3DT1TFE can be used to systematically identify and quantify these deeply hypointense foci.*

• *Deep T1-hypointensity may act as an easily detectable, surrogate marker for PRLs.*

**Graphical Abstract:**

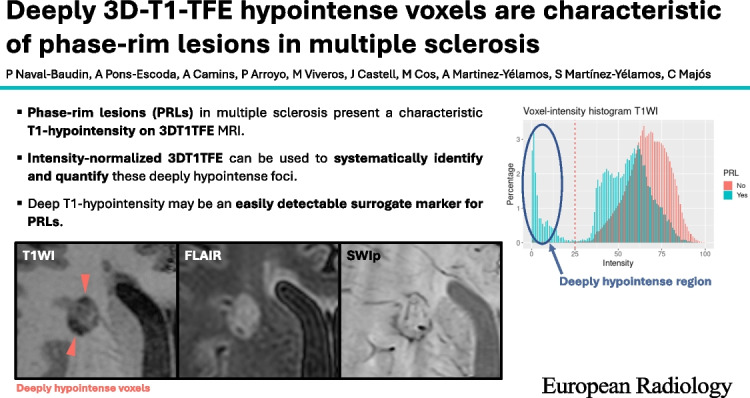

## Introduction

### Background

Multiple sclerosis (MS) is a neurodegenerative and inflammatory demyelinating disease that represents one of the leading causes of disability in young adults. The clinical course of MS is heterogeneous, ranging from benign forms with little permanent clinical impact to rapidly disabling progressive forms [1]. In recent decades, several highly effective disease-modifying drugs have been developed, primarily for the treatment of relapsing–remitting MS (RRMS) [2]. As a result, acute disease relapse has long been the main focus of MS clinical and imaging monitoring.

However, recently approved treatments have been shown to slow disability progression in patients with secondary progressive MS (SPMS) [2, 3]. Given this new opportunity to effectively treat patients with SPMS, it is more crucial than ever to detect transformation to progressive disease as early as possible [4]. Several imaging findings have been proposed as potential markers for progressive disease, including phase-rim lesions (PRLs) and slowly expanding lesions (SELs), among others [5–9]. These two imaging signs seem to be correlated with each other, to correspond to chronic smoldering inflammation [8], and their presence is associated with a worse clinical prognosis [6–11]. In turn, these areas of smoldering inflammation appear to be correlated with lesions that are hypointense on T1-weighted imaging (T1WI; “T1 black holes”) [5, 8, 12, 13]. However, neither PRLs nor SELs are easily detected on standard follow-up magnetic resonance imaging (MRI) in MS [7]. Moreover, identifying PRLs is not overly straightforward; compared to fluid-attenuated inversion recovery (FLAIR)-hyperintense or enhancing lesions, PRLs show very subtle lineal hypointensity similar to small vascular structures. Furthermore, their quantification is not easily amenable to inclusion in standard radiological workflows. Specifically, visualization of PRLs requires the incorporation of susceptibility-weighted imaging (SWI) in imaging protocols; however, this imaging modality is not routinely recommended in standard follow-up MRI protocols for MS patients [14, 15]. Even if SWI is available, these lesions are most commonly described on 7 T MRI, and to a lesser degree on 3 T [10, 16, 17] and 1.5 T MRI [18]. They are also much less conspicuous than FLAIR-hyperintense and contrast-enhancing lesions.

Previous studies have described a characteristic T1 hypointensity of these smoldering lesions [11]. In fact, in the authors’ clinical experience, PRLs often correspond to lesions that are deeply hypointense on T1WI. This phenomenon is particularly prominent on routine 3D T1 inversion recovery-gradient echo sequences, such as 3DT1TFE (Philips), MPRAGE (Siemens), 3D IR-SPGR, BRAVO (General Electric), and 3D Fast FE (Canon) [19]. The appearance of these lesions is distinct from that of non-phase-rim non-enhancing FLAIR-hyperintense white-matter lesions (nPR-WMLs).

### Objectives

We sought to compare the intensity profiles of PRLs on 3 T 3DT1TFE MRI to those of nPR-WMLs and to analyze the lesion-classification potential of these T1-intensity profiles as surrogates for identifying PRLs.

## Materials and methods

### Study approval

The present study was reviewed and approved for publication by the Research Ethics Committee of Bellvitge University Hospital. Patient data were anonymized and confidentiality was maintained in accordance with national and European Union regulations. A nonspecific informed consent for participation in research projects was obtained from all patients and the ethics committee waived the requirement for specific informed consent for this retrospective study.

### Study design and recruitment

For this retrospective observational cross-sectional study, subjects were identified for inclusion from our radiology department registry of MRIs ordered by our center’s MS department between August 1, 2019, and June 9, 2020. Eligibility criteria included the following: (1) subjects diagnosed with MS, (2) a 3 T MRI had been acquired by our center during the accrual period, and (3) the available MRI sequences included 3DT1TFE images acquired before and after intravenous gadolinium administration, 3D FLAIR images, and SWI. A balanced sample of 10 SPMS subjects was then consecutively obtained from the eligible candidates and then matched with another 10 RRMS subjects by age at MRI acquisition, sex, and time since disease onset.

### Imaging

All MRI studies were performed with the same Philips Ingenia 3 T scanner using either a 16- or 32-channel head coil. Sequence acquisition parameters were as follows: 3DT1TFE—TE: 4.9 ms; TR: 10 ms; flip angle: 8°; matrix: 512 × 512; slice thickness: 1 mm; in-plane resolution dimensions: 0.46 × 0. 46 mm; 3D FLAIR—TE: 309 ms; TR: 5500 ms; flip angle: 40°; matrix: 512 × 512; slice thickness: 1.1 mm; in-plane resolution: 0.49 × 49 mm; SWI-phase (SWIp)—TE double echo: 7.2 ms and 13.4 ms; TR: 31 ms; flip angle: 17°; matrix: 768 × 768; slice thickness: 1.2 mm; in-plane resolution: 0.3 × 0.3 mm. Of note, Philips SWIp internally uses a proprietary method to fit multiple echo images and generate a single image magnitude/phase image pair to improve signal to noise ratio [20]. Intravenous contrast (gadobutrol: 1 mmol/mL, 0.1 mmol/kg) was administered with a delay of at least 5 min before contrast-enhanced T1WI acquisition.

### Image pre-processing

Images were converted from DICOM to NifTI format with dcm2niix using the MRIcroGL suite (https://nitrc.org/projects/mricrogl) [21]. Steps requiring FSL tools were performed on version 6.0.5 (https://fsl.fmrib.ox.ac.uk/) [22]. Image reorientation, cropping, initial brain extraction, and bias field correction were performed with the structural image processing pipeline “fsl_anat.” Non-contrast T1W images were resliced to isotropic 0.5-mm images with the FMRIB Linear Image Registration Tool (FLIRT) [23] and used as a reference space in which a new brain mask was obtained with the “optiBET” script (https://montilab.psych.ucla.edu/fmri-wiki/optibet/) [24]. The rest of the sequences were rigidly co-registered to the T1WI reference space with FLIRT and the non-contrast T1-acquired brain mask was applied to them. Finally, intensity normalization was performed on T1W images with and without contrast using the Piecewise Linear Histogram Matching method reported by Nyul and Udupa [25] and included in the “intensity-normalization” package (https://intensity-normalization.readthedocs.io) [26, 27] in Python version 3.8.10. For reproducibility purposes, the group histogram normalization templates for the pre- and post-contrast T1WIs used in this study are available upon request to the corresponding author and can be applied to pre-processed images.

### Lesion segmentation

**Fig. 1:**
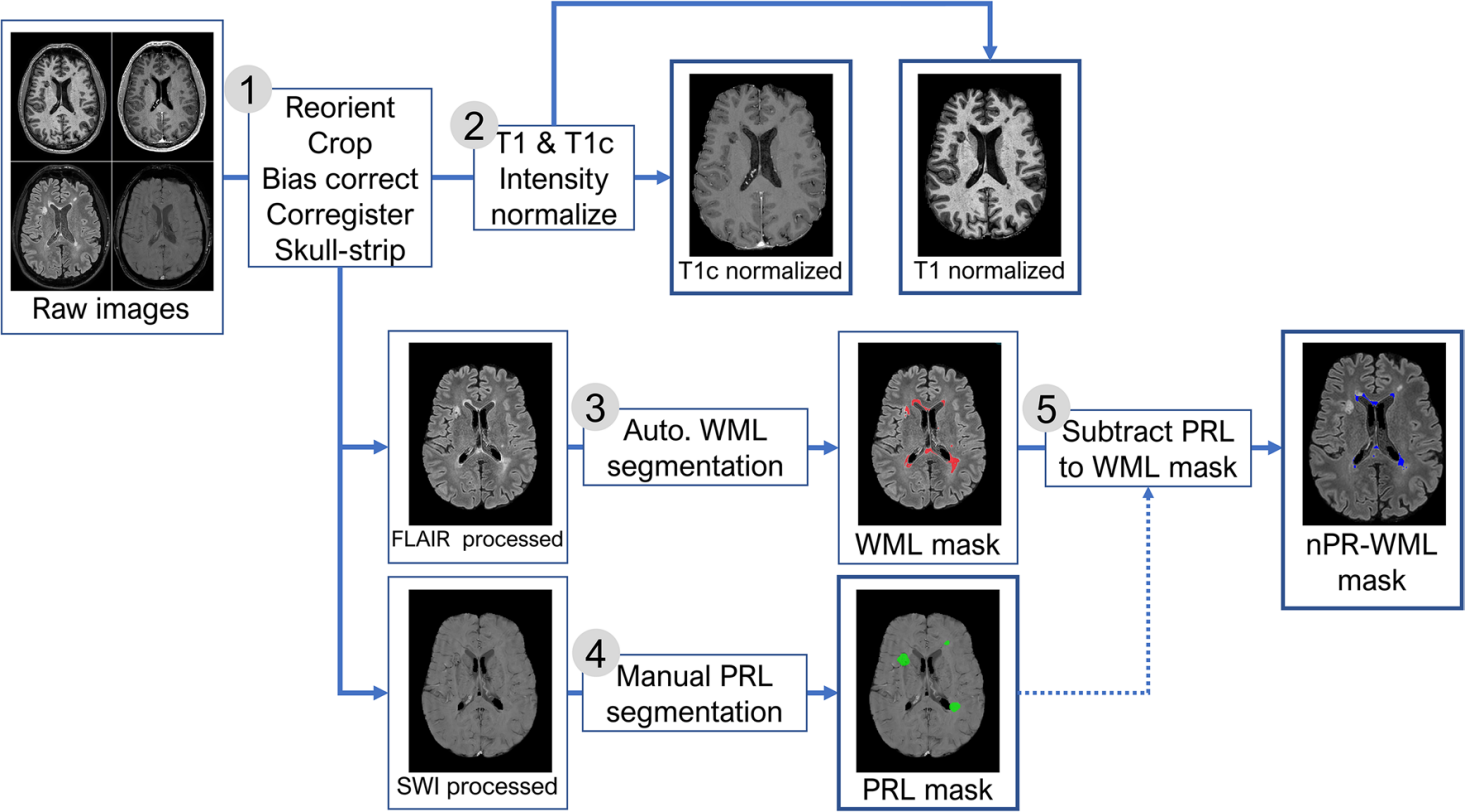
Image pre-processing and segmentation workflow. Abbreviations: 3DT1TFE without contrast (T1), 3DT1TFE with contrast (T1c), fluid-attenuated inversion recovery (FLAIR), susceptibility-weighted imaging (SWI), white-matter lesion (WML), phase-rim lesion (PRL), non-phase-rim white matter lesion (nPR-WML)

The image pre-processing and segmentation workflow is presented in Fig. 1. PRLs were manually segmented on the SWI images with ITK-SNAP (http://itksnap.org/) [28] by a neuroradiologist with 4 years of experience (P.N-B.). The corresponding FLAIR images were viewed concomitantly for support. Automatic white-matter lesion segmentation was performed on 3D FLAIR images with the lesion prediction algorithm implemented in the LST toolbox version 3.0.0 (https://statistical-modelling.de/lst.html) [29] for SPM 12. Probability lesion maps were binarized with a lower threshold of 0.5 to establish a total FLAIR lesion map. PRL segmentations were subtracted from the total FLAIR lesion map to establish two complementary final segmentation maps of (1) PRLS and (2) nPR-WMLs. Finally, individual lesions from either map were then isolated into individual single-lesion binary mask files. These thresholding, arithmetic, and clustering image operations were performed using the “fslmaths” and “cluster” functions available in FSLUTILS.

### Data extraction

Demographic and clinical data obtained from our MS unit’s database included age, sex, and time since disease onset. Voxel-wise data were obtained with the “oro.nifti” [27] package for R version 3.6.3 and lesion-wise data were obtained with the “fslstats” function in FSLUTILS [22]. These data included (1) the total number and volume of PRLs and nPR-WMLs, (2) the volume of each lesion on MRI, (3) the voxel intensity values of the combined PRLs and combined nPR-WMLs on T1WI, and (4) the fifth percentile (p5) T1WI intensity value of each lesion.

### Data analysis

All data analyses were performed with R version 3.6.3. For analysis of demographic and MRI lesion data, Fisher’s exact test was used for categorical variables and the Wilcoxon rank-sum test was used for quantitative variables. For voxel-wise analysis, voxel intensity values for nPR-WMLs and PRLs were depicted with histograms. Due to the expected sample imbalance, histogram bar height represents the percentage of voxels for each group instead of frequency. For lesion-wise analysis, all lesions under 1 mm^3^ and those with mean normalized intensities greater than 80 on T1WI with contrast were discarded to avoid including contrast-enhancing lesions; these lesions were ruled out to avoid non-contrast T1 hypointensity due to acute inflammation. In the final sample of non-enhancing lesions, contrast analyses of PRL and nPR-WML p5 intensities were performed with the Wilcoxon rank-sum test.

For lesion classification analysis, the sampled lesions were split equally (50%) into training and test subsets. A lesion classifier was created taking a single variable: the p5 normalized intensity of each lesion on T1WI. The p5 normalized intensity refers to the 5th percentile intensity value of the normalized T1-intensity histogram of each lesion. The aim was to explore the hypointense-most voxels in each lesion. The p5 intensity value was chosen as it was considered the optimal point which allowed to analyze the lower limits of the intensity histograms while avoiding extreme values which might be outliers. The optimal receiver-operator curve cutoff point in the training set was identified automatically using the “cutpointr” R package. This package selects a cutoff point for ROC curves based on prioritized metrics; in this case, specificity was prioritized by specifying the “spec_constrain” option. A highly specific cutoff point was necessary due to the expected severe sample imbalance with PRLs being much less frequent than nPR-WMLs. A minimum decrease in test specificity would result in a large increment in false positive results. The selected cutoff point was applied to the test set for lesion classification and the resulting confusion matrix was analyzed with Fisher’s exact test.

## Results

### Participants and lesions

Twenty subjects were included in our study (10 RRMS and 10 SPMS patients). Automatic white-matter lesion segmentation detected a total of 2569 lesions, including 1408 among RRMS patients and 1161 among SPMS patients. However, the total lesion volume was smaller in RRMS patients than in SPMS patients (75.6 cm^3^ vs. 140.2 cm^3^). Manual PRL segmentation retrieved 12 lesions in 5 of the 10 RRMS patients (8 of them in a single patient) and 27 lesions in 9 of the 10 SPMS patients. Refer to Table 1 for further details on demographic and MRI lesion data.

**Table 1:** Demographic and summary MRI data. Data is presented as “median (range)” except for Gender and PRL present, which are presented as “subject count (percentage).” Years since onset refers to time elapsed since diagnosis. Global WML refers to all WMLs detected by automatic segmentation, regardless of phase-rim status. Significance statistics contrast groups RRMS and SPMS. Wilcoxon rank-sum test for quantitative variables. Fisher exact test for binary variables “Gender” and “PRL present”

	All subjects (*n* = 20)	RRMS subjects (*n* = 10)	SPMS subjects (*n* = 10)	*p*
Age [years]	51.5 (39–62)	50.5 (46–62)	52.5 (39–61)	0.85
Gender [male]	6 (30%)	3 (30%)	3 (30%)	1
Years since onset	20 (6.7–32.3)	18.05 (6.7–32)	21.5 (7.6–32.3)	0.73
PRLs present	14 (70%)	5 (50%)	9 (90%)	0.14
PRL count	1 (0–8)	0.5 (0–8)	2.5 (0–6)	0.02*
PRL volume [cm^3^]	.25 (0–7.43)	.078 (0–7.43)	.62 (0–4.12)	.07
Global WML count	90.5 (64–428)	101.5 (64–428)	116 (73–237)	0.79
Global WML volume [cm^3^]	10.78 (2.16–25.63)	6.49 (2.16–17.78)	15.29 (3.72–25.63)	0.04*

*RRMS* relapsing–remitting multiple sclerosis, *SPMS* secondary-progressive multiple sclerosis, *PRL* phase rim lesion, *WML* white matter lesion

^*^p value < 0.05

### Voxel-wise analysis

**Fig. 2:**
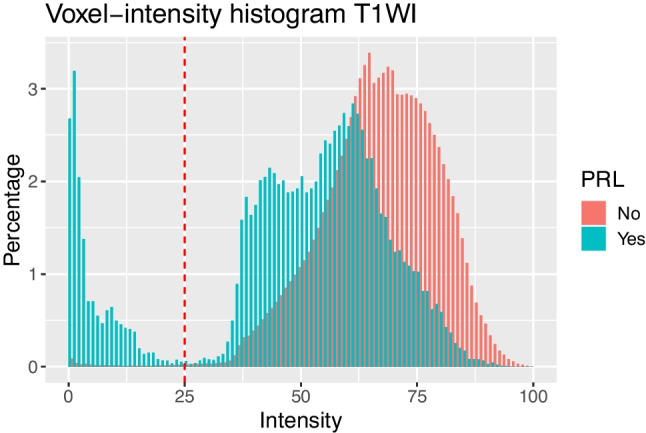
Overlaid voxel-intensity frequency percentage histograms of phase-rim lesions (blue) and non-phase-rim white-matter lesions (red). Vertical red line represents the minimum frequency between the two modes

Voxel-wise intensity histograms (Fig. 2) demonstrated a unimodal nPR-WML distribution with a maximum normalized intensity frequency at 64.8. However, PRLs displayed a bimodal intensity distribution, with the two modes showing normalized intensity frequencies of 0.9 and 60.2. The minimum frequency point between the two modes was at intensity 25. Binary stratification of voxels at this cutoff point revealed an over-representation of deeply hypointense voxels in the PRL sample. Specifically, approximately 14% of PRL voxels were located in the deeply hypointense segment in contrast to only 0.5% of nPR-WML voxels.

### Lesion-wise analysis

**Fig. 3:**
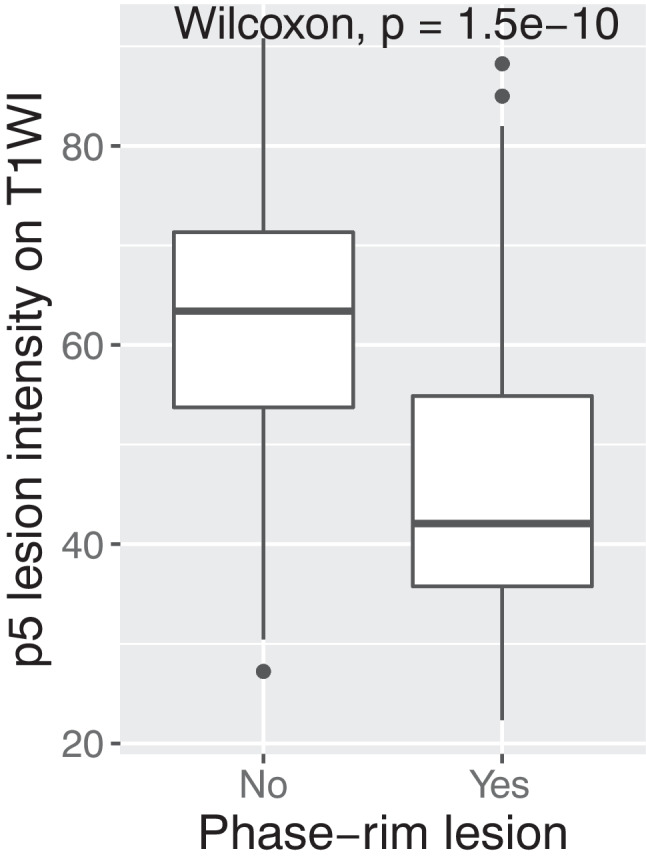
Boxplot representation of the distribution of non-phase-rim white-matter lesions vs. phase-rim lesions

Automatic lesion segmentation and manual extraction of PRLs from the automatic lesion classification maps yielded a total of 2620 lesions, of which 39 were PRLs and 2581 were nPR-WMLs. For lesion-wise analysis and lesion classification, all lesions under 1 mm^3^ and with intensities greater than 80 on post-contrast T1WI on baseline MRI were discarded. Thus, a total of 1075 nPR-WMLs and 39 PRLs remained. Analysis of the p5 T1WI intensity values of individual PRLs vs. nPR-WMLs revealed a significantly lower value for PRLs (Fig. 3).

### Lesion classification

**Fig. 4:**
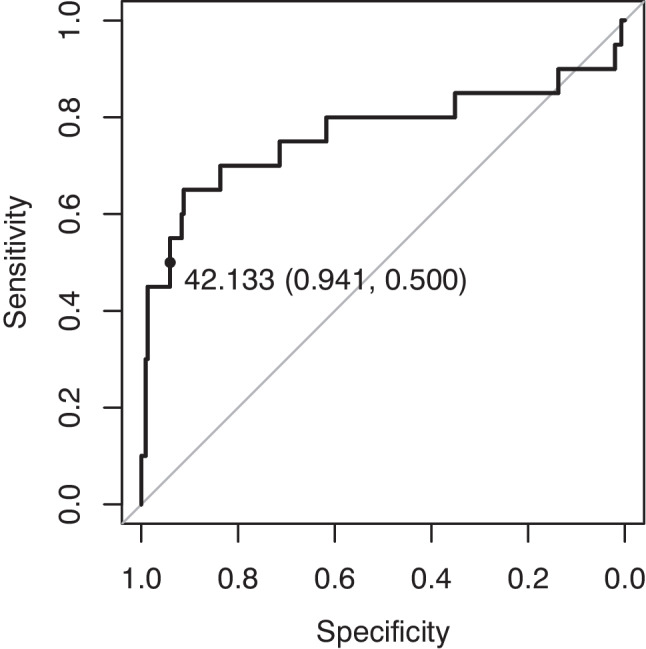
Receiver-operator curve of the fifth percentile of the non-contrast T1WI normalized intensity value of each lesion, enabling the classification of phase-rim and non-phase-rim white-matter lesions in the training set

**Fig. 5:**
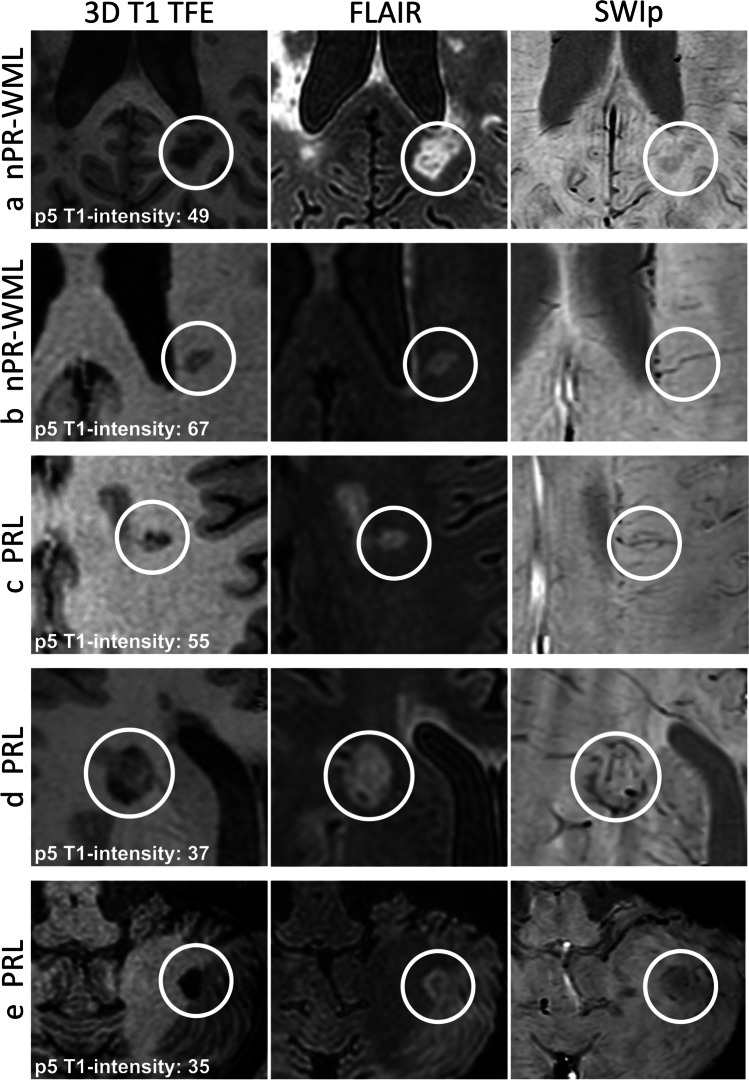
Lesion examples. The top two rows (**a**–**b**) show non-phase-rim white-matter lesions (nPR-WMLs). The bottom three rows (**c**–**e**) show phase-rim lesions (PRLs). The fifth percentile-normalized T1 intensity of each lesion (p5 T1 intensity) is specified in the first column. Lesion (**a**) is a nPR-WML with areas of marked T1 hypointensity. Lesion (**b**) is a nPR-WML with mild T1 hypointensity. Lesion (**c**) is a PRL with small, peripheral, very T1-hypointense foci. Lesions (**d**, **e**) are PRLs with profound T1-hypointense rims. Note that lesion (**c**) falls above the classification threshold (42.133) and would therefore correspond to a false negative. Also note that lesion (**a**), which is a nPR-WML, shows areas of prominent visual T1 hypointensity but is quantitatively above the threshold, thereby corresponding to a true negative

A single-variable lesion classifier was created based on the p5 normalized intensity value of each lesion on non-contrast T1WI (Fig. 4). The training subset of lesion-wise p5 intensity values included 20 PRLs and 538 nPR-WMLs. The test subset included 19 PRLs and 537 nPR-WMLs. The automated optimal cutoff point for the classifier was at a T1 intensity value of 42.133. Application of this cutoff point to the test set yielded a sensitivity of 0.526, specificity of 0.959, positive predictive value of 0.313, negative predictive value of 0.982, and F1-score of 0.392 for PRL detection. Examples of PRLs and nPR-WMLs with their corresponding p5 T1WI intensity values are presented in Fig. 5.

## Discussion

In this study, we found that PRLs tended to contain deeply hypointense voxels on 3DT1TFE and that these markedly hypointense voxels were practically absent in nPR-WMLs. We also demonstrated that these differences can be explored on a lesion-wise basis by analyzing the lower percentile range of the normalized T1WI intensity of each lesion. Lesion classification based on the lesion-wise p5 intensity was very specific for PRL detection (specificity: 0.959).

The advent of disease-modifying therapies for progressive MS underscores the need for biomarkers for the early and accurate identification of treatment candidates [2, 3]. PRLs have previously been identified as putative biomarkers. For example, Absinta and colleagues demonstrated that PRLs were associated with less lesion-volume shrinkage than nPR-WMLs and became progressively more T1-hypointense over time. Autopsy reports then showed that PRLs corresponded to iron-laden inflammatory myeloid cells at the edges of lesions [8]. In a subsequent study, they prospectively analyzed 2019 patients with 3 T or 7 T MRI, 56% of which had PRLs. They found that patients with 4 or more PRLs reached disability status sooner than those with 3 or fewer; that PRLs tended to expand over time, unlike nPR-WMLs which tended to shrink; and that PRLs were more hypointense on T1WI, suggesting more profound tissue damage [9]. More recently, Treaba and colleagues showed that PRL count and volume on 7 T MRI were predictors of neurological disability progression in MS patients along with normalized subarachnoid cerebrospinal fluid volume, leukocortical lesion volume, and normalized white-matter volume, among other features [7]. These results are consistent with the hypothesis that PRLs and SELs represent smoldering or chronic inflammation, which would be more prevalent in progressive MS than in RRMS [6, 7, 10, 11]. Despite the promising results of this previous work, however, there are several reasons why these putative biomarkers are unlikely to be introduced into clinical workflows in the near future. First, evaluation of these biomarkers requires SWI sequences, and international guidelines do not include this imaging modality in standard MS MRI follow-up protocols [14]. Second, these biomarkers have been described chiefly in higher-magnetic field 7 T magnets and to a lesser degree on 3 T and 1.5 T scanners which are more widely available [10, 16–18]. Finally, and most importantly, PRL identification is not straightforward and requires meticulous image examination. Thus, it is challenging to routinely quantify PRLs in clinical practice.

Nevertheless, our results herein demonstrate that 3DT1TFE MRI may be a novel screening method for PRLs. The identification of a profoundly hypointense lesion on this sequence may signal the possibility of a PRL and direct the radiologist to search for rim lesions on SWI. Moreover, in specific clinical scenarios, in the absence of SWI availability, profoundly hypointense lesions on 3DT1TFE could be considered a surrogate of PRLs. T1WI, either with or without contrast, is much more readily available than SWI and is routinely included in MRI brain scans irrespective of magnetic field type. Moreover, deeply T1WI hypointense foci are visible to the radiologist’s eye and are much more straightforward to objectively quantify by intensity histogram analysis than the presence of PRLs on SWIp.

The T1WI hypointensity of PRLs has been previously described in the literature [5, 8, 12, 13]. For example, a recent paper by Kee Kwong and colleagues demonstrated that 100% of PRLs (*n* = 25) identified in 45 MS patients corresponded to T1-hypointense lesions [13]. However, our study is the first to thoroughly and quantitatively analyze the normalized 3DT1TFE hypointensity of these lesions and propose its feasibility as a surrogate marker of smoldering inflammation. Furthermore, these findings suggest that PRLs are the sites of profound ongoing tissue damage.

The present study has several limitations. First is the use of non-quantitative imaging. Specifically, although our study is centered on intensity quantification, non-quantitative T1 imaging was performed. Nevertheless, these image sequences are the most widely available because they are recommended in clinical MS imaging guidelines [14, 15]. Therefore, to compensate for the shortcomings of this approach, we implemented an intensity normalization method that is commonly used in the literature [26]. Nevertheless, suboptimal normalization can introduce random noise to the data, thereby hindering analyses. The second limitation is that PRLs have often been described in the literature in the context of very high-field 7 T MRI scanners. Thus, our analysis of 3 T MRI scans may limit the number of PRLs that we can detect. However, the optimal MRI scanners available at most clinical centers worldwide are 3 T devices [14]. Third, our study is limited by its small patient sample size (20 subjects). However, this sample is homogeneous and of high quality; all images were performed on a 3 T MRI scanner following an identical protocol, in line with international guidelines. Furthermore, we performed voxel-wise and lesion-wise analyses rather than patient-wise analyses. Therefore, the smallest group analyzed contained 39 PRLs. In summary, we consider this a proof-of-concept study. Future research should consist of a multicentric analysis including a more varied array of image sequence parameters. Finally, we want to clarify that we refrain from making assumptions about patient-specific results in this paper, as the primary objective is not to investigate individual patient outcomes, for which a larger patient sample would be necessary.

In conclusion, this study demonstrated that deep T1WI hypointensity lesions on 3DT1TFE MRI are relatively specific for PRLs. These preliminary results suggest that these lesions could serve as surrogate markers for PRLs, paving the way for improved study of smoldering lesions and progressive MS activity. Moreover, the availability of T1WI, together with the straightforward evaluation and quantification of lesion intensity, renders it more amenable to clinical implementation than SWI-based PRL detection. Future studies might aim at confirming the reproducibility of these findings in larger subject samples and with different MRI acquisition parameter configurations, as well as exploring other methods of quantification and standardization of deep T1WI hypointensity and evaluating its prognostic value.

## Abbreviations

3DT1TFE: Three-dimensional T1-weighted turbo field echo
FLAIR: Fluid-attenuated inversion recovery
MRI: Magnetic resonance imaging
MS: Multiple sclerosis
nPR-WMLs: Non-phase-rim white-matter lesions
P5: Fifth percentile
PRL: Phase-rim lesion
RRMS: Relapsing-remitting MS
SEL: Slowly expanding lesion
SPMS: Secondary progressive MS
SWI: Susceptibility-weighted imaging
SWIp: Susceptibility-weighted imaging phase
T1W1: T1-weighted imaging

## Declarations

### Guarantor

The scientific guarantor of this publication is Pablo Naval-Baudin.

### Statistics and biometry

One of the authors has significant statistical expertise.

### Study subjects or cohorts overlap

None of the study subjects have been previously reported.

### Methodology

• Retrospective cross-sectional study and diagnostic study performed at one institution.
